# Pain, fatigability and cognitive impairment in log-COVID: a cohort study

**DOI:** 10.1192/j.eurpsy.2024.765

**Published:** 2024-08-27

**Authors:** B. Nadia, T. Mariem, B. A. Houda, E. Sahar, A. Wissal, M. Sameh, K. Samy, H. Najla, A. Jihen

**Affiliations:** ^1^Psychiatry “B”; ^2^Preventive medecine and hospital hygiene; ^3^Pneumology, Hedi Chaker university hospital, Sfax, Tunisia

## Abstract

**Introduction:**

Survivors of the pandemic of COVID-19 suffered from multiple sequelae long time after recovery, such as tiredness and memory dysfunction, affecting daily life activities.

**Objectives:**

To assess fatigability, cognitive impairment and the severity of pain in long-COVID.

**Methods:**

We conducted a prospective cohort study including 121 Tunisian COVID-19 inpatients who had been discharged alive from hospital. Each enrolled patient was asked about the period before the hospital stay, and the 6-9 month-period after hospital discharge, using *the visual analog scale* (*VAS*), *self-completed* unidimensional scale and yes/ no question about fatigability and cognitive impairments.

**Results:**

The median age of participants was 59 years, with extreme values ranging from 18 to 80. Among them, 51.2% were females.

Our findings showed a significant increase in VAS score after COVID infection (3.82 vs 1.69; p<0.001). Sixty-eight (56.2%) participants reported spontaneously fatigability after the infection and 52 (43%) reported spontaneously a deterioration in memory capacity either with or without previous memory dysfunction. Fatigability was statistically associated to cognitive impairment (55.9% vs 26.4%; P= 0.02). In addition, fatigability and cognitive impairment were statistically associated with pain (P=0.001 and P= 0.022 respectively).

There was no significative association of fatigability nor cognitive impairment with the gender of the survivors.

**Conclusions:**

The clinician should keep in mind to screen for possible somatic or psychological distress, in particular pain, fatigability and cognitive impairment even after resolution of the COVID infection, in order to guarantee a better quality of life.

**Disclosure of Interest:**

None Declared

